# Rhinosporidiosis- Epidemiological, Clinicoradiological, Immunological Profile

**DOI:** 10.22038/IJORL.2023.68378.3331

**Published:** 2023-09

**Authors:** Megha Chandran, Rupa Mehta, Nitin M Nagarkar, Anudita Bhargava, Eli Mohapatra, Saroj Kumar Pati

**Affiliations:** 1 *Department of ENT-Head and Nech Surgery, AIIMS Raipur, India.*; 2 *Department of Microbiology, AIIMS Raipur, India.*; 3 *Department of Biochemistry, AIIMS Raipur, India.*; 4 *Department of Radiology, AIIMS Raipur, India*

**Keywords:** Endemic, Cell-mediated immunity, Cytokines, IL-6, TNF-beta, IFN-gamma

## Abstract

**Introduction:**

Rhinosporidiosis is an enigmatic disease with many unsolved queries right from taxonomy to treatment. This study has been done to understand the disease characteristics with a peek into the lesser known immunological aspects of it by studying the changes in levels of certain primarily cell-mediated immunity (CMI)-specific cytokines in rhinosporidiosis patients.

**Materials and Methods:**

A prospective observational study was performed. Detailed epidemiological and clinicoradiological assessment was done along with selected inflammatory and immunological markers. The tests for immunological parameters were done by ELISA and CLIA and data were compiled and analyzed using appropriate statistics.

**Results:**

Disease showed male predominance and all patients gave a universal pond bathing history. Majority patients had O+ve blood group. Right side was affected most with nasal obstruction being commonest symptom. Nasal cavity was involved in majority of cases with inferior turbinate and meatus being sites of maximum occurrence and attachment. Nasopharynx, oropharynx were other involved sites. Extra-nasal sites included skin and parotid gland. Endoscopic and CECT findings were similar and confirmed intraoperatively. Tests for inflammatory markers showed no significant change in patients. Immunological markers -IL-6, TNF-beta- levels showed significant increase though no such increase was found with IFN-gamma levels.

**Conclusion:**

Rhinosporidiosis has a definite epidemiological and clinical-radiological profile. A clear association with exposure to contaminated water is present which could not be further associated with disease duration or recurrence. The immunological profile needs to be further investigated upon since it remains quite elusive.

## Introduction

Rhinosporidiosis is a chronic granulomatous condition caused by *Rhinosporidium seeberi*, an aquatic protozoan belonging to class Mesomycetozoea (1). It is a disease that involves mucosal and subcutaneous tissue (2). 

Rhinosporidiosis is an endemic disease found in tropical regions, most prevalent in India (South and Central India) (3), Sri Lanka, and Argentina. It also occurs in North America, Africa, Europe, and Asia. Most cases in the western world occurred among immigrant population who acquired disease in their endemic native land (4).

Mode of infection is transepithelial by inoculation of traumatized epithelium with sporangiospores (5). On exposure to infected water (ponds, lakes) or soil, the spores penetrate nasal cavity mucosa where they mature into sporangium within submucosa; after maturation, sporangia burst, releasing endospores into surrounding tissue causing typical lesions, characterized by reddish, friable, pedunculated, soft tissue polypoidal ‘strawberry-like” masses studded with white spots (mature sporangia).

Mucous membranes of upper respiratory sites are commonly affected especially nose & nasopharynx. Extra-nasal sites oropharynx, larynx, trachea, conjunctiva, lacrimal sac are also affected along with, other sites like lips, palate, buccal mucosa, genital mucosa, skin, scalp and bone (5). 

*Rhinosporidium seeberi *defies Koch’s postulates. It has so far neither been cultured in vitro nor has been proven to be pathogenic to the experimental animals tested. This is a major limitation in studying and amassing information on the immune responses of the organism. So far it is cell mediated immunity that has been found to have a greater role in immune reactions in rhinosporidiosis. 

The immunologic characteristics of rhinosporidiosis is a less trodden aspect of the disease which we have tried to explore further through our study. Although many aspects of the disease like its chronicity, severity and dissemination suggest a definite host immunological response, there is no conclusive evidence in literature regarding association with specific cytokines or immune markers. 

In our study, we have tried to find any association the disease might have with common broad-spectrum cytokines thus shedding light on immunological aspect of this disease.

*Objectives*

 To study epidemiological, clinico-radiological profile and changes in levels of certain primarily cell-mediated immunity (CMI)-specific cytokines [Tumour Necrosis Factor-beta (TNF-β), Interferon-gamma (IFN-γ)] and Interleukin-6 (IL-6)] in cases of rhinosporidiosis, in a tertiary health care center in Chhattisgarh.

## Materials and Methods

A prospective observational study of 6 months duration was conducted in a tertiary healthcare center in Raipur. 

After approval from Institutional Ethics Committee, all clinically diagnosed cases of rhinosporidiosis operated in the department of ENT and Head – Neck surgery during the study period of 6 months were included in the study based on appropriate inclusion and exclusion criteria. Radiological (Contrast-enhanced CECT) assessment was done for all patients. A well-informed written consent was taken. 

a) Inclusion criteria:

i. Clinically diagnosed cases of Rhinosporidiosis during the study period (patients were enrolled in the study and analysis after clinical examination and later diagnosis was confirmed with histopathological report).

b) Exclusion criteria:

i. Patients with nasal mass with a diagnosis other than rhinosporidiosis.

ii. Patients with any immunosuppressive conditions (Immunodeficiency diseases, patients on long term steroid therapy, etc.) or autoimmune disorders (for example, rheumatoid arthritis, lupus, etc.) that are likely to affect the immunological profile).

iii. Patient who refused to give consent.

iv. Pregnant patients.

**Fig 1:**
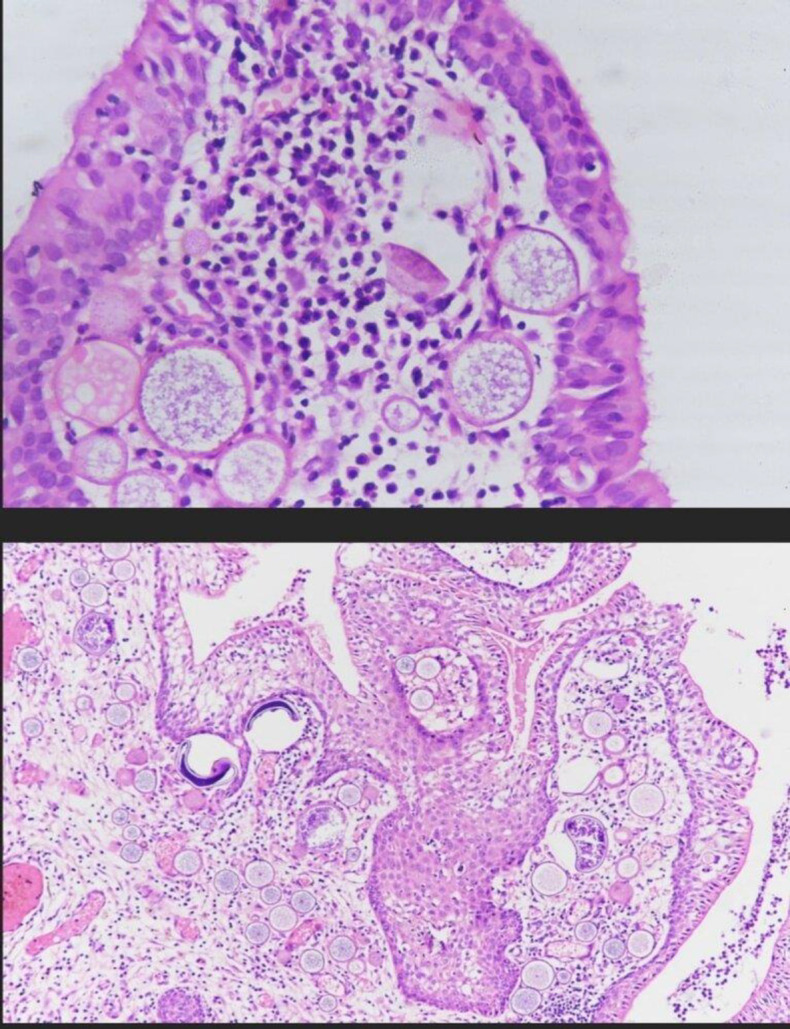
Rhinosporidiosis histopathology slides- H&E stain

Histopathological confirmation was obtained from the pathology department for all specimens using rapid Hematoxylin and Eosin (H&E) and Periodic acid Sciff (PAS) staining. Smears revealed multiple globular sporangia filled with spores (stain magenta on PAS- PAS positive) and inflammatory cells in fibrous stroma (Figure 1).

Tests for Inflammatory markers – Erythrocyte Sedimentation Rate(ESR), C-Reactive Protein(CRP), Ferritin – and Immunological markers – Interleukin-6 (IL-6), Tumor Necrosis Factor-beta(TNF-β), Interferon-gamma(IFN-γ) –were done for the patients (results of IL-6, TNF- β and IFN- γ were compared with that of age and sex matched disease-free individuals in a 3:1 ratio). The tests for immunological markers were conducted in the Department of Biochemistry and Department of Microbiology. Serum samples from patients and healthy individuals (one-third the number of study subjects) were collected and stored at -80^0^ Celsius. Further processing was done using following immunological methods after complete sample collection.

IL-6 –Chemiluminescence Immunoassay (CLIA) using ADVIA Centaur IL6 assay.

TNF- β/ IFN-γ – Enzyme-linked Immunosorbent Assay (ELISA). 

Following data collection, analysis was done using SPSS 21version (IBM Corp.). Group comparisons were done using independent sample ‘t’ test for continuously distributed data and appropriate nonparametric tests in the form of Wilcoxon Test in non-normally distributed data. Chi-squared test was used for group comparisons for categorical data. In case the expected frequency in the contingency tables was found to be <5 for >25% of the cells, Fisher’s Exact test was used instead. Linear correlation between two continuous variables was explored using Pearson’s correlation (normally distributed data) and Spearman’s correlation (non-normally distributed data). Statistical significance was kept at p < 0.05.

## Results

A total of 31 patients were included in the study of which 26 were males and 5 were female patients. Maximum number of patients belonged in the age group 10-20 years. 

All patients gave history of pond bathing, 96.8% of the participants gave a positive history of animal bath in the same pond and 58% gave a history of agriculture/cattle exposure. However, there was no significant association found between duration or frequency of pond-bathing with disease characteristics like recurrence in our study. Only 2 patients (6.5%) had awareness about the disease association with pond bathing while the rest 93.5% were ignorant about the risk. 

Disease was found to affect O+ve blood group individuals the most. 

61.2% patients were new cases while 38.8% patients came with recurrent disease. 

Right side was commonly involved (17 cases/54.8%) and most common symptoms were nasal obstruction, visible mass in nasal cavity and epistaxis. All the 30 nasal- nasopharyngeal- oropharyngeal cases of rhinosporidiosis were pedunculated. 3 cases (9.7%) had single stalk; others had multiple stalks with attachment to various sites.

On endoscopic assessment, the most common site (77.4%) of attachment of stalk of rhinosporidiosis was seen in the inferior turbinate followed by the inferior meatus (45.2%) with 1 case having found to have attachment at the nasolacrimal duct opening(25.8%). Other nasal cavity sites included septum (25.8%), vestibule (3.2%), middle meatus (3.2%) and floor (12.9%). Among the nasopharynx sites, the most common attachment of stalk was to the posterior pharyngeal wall (9.7%) and choana (9.7%). Oropharyngeal sites included posterior pharyngeal wall (3.2%), posterior pillar (3.2%) and soft palate (3.2%). Stalk attachment to the tongue was found in 1 patient (3.2%) Extra nasal sites involved nasopharynx, oropharynx, oral cavity, lacrimal apparatus and maxillary sinus. Cutaneous involvement was found in 6.7 % with concurrent nasal-nasopharyngeal-oropharyngeal disease. One case (3.2%) had isolated parotid ductocele secondary to rhinosporidiosis of parotid duct. 

Lesion over tongue, lacrimal sac disease and paranasal sinus involvement (maxillary sinus) was found in 1 patient (3.2%) each. Cutaneous involvement was found in 2 cases (6.7%) which included well-defined firm swelling in left arm and left forehead in one and bleeding ulcerative lesions in the right forearm, right leg and left ankle in the other. Both the cases had nasal- nasopharyngeal- oropharyngeal involvement also. Inflammatory markers (ESR, CRP, Ferritin) were not found to have a significant change or increase in patients. 

Immunological markers (IL-6, TNF-β, IFN-γ) levels in comparison with healthy controls showed a significant increase in IL-6 and TNF-β levels in patients (p-value <0.05). Chi-squared test was used to explore the association between Il-6 Levels of cases and controls. There was a significant difference between case and control groups in terms of distribution of Il-6 Levels (χ2 = 33.472, p = <0.001) (Table 1). 

**Table 1:** Association Between case and control groups and Il-6 Levels (n = 45)

**Il-6 Levels**	**Group**			**Chi-Squared Test**	
	**Case**	**Control**	**Total**	**χ2**	**P Value**
<10.3	3 (9.7%)	14 (100.0%)	17 (37.8%)	33.472	<0.001
≥10.3	28 (90.3%)	0 (0.0%)	28 (62.2%)		
Total	31 (100.0%)	14 (100.0%)	45 (100.0%)		

9.7% of the patients had Il-6 Levels: <10.3, while 90.3% of the cases had Il-6 Levels: ≥10.3 All controls had Il-6 Levels: <10.3. With a p-value <0.001 it shows the association to be significant.

**Graph 1:**
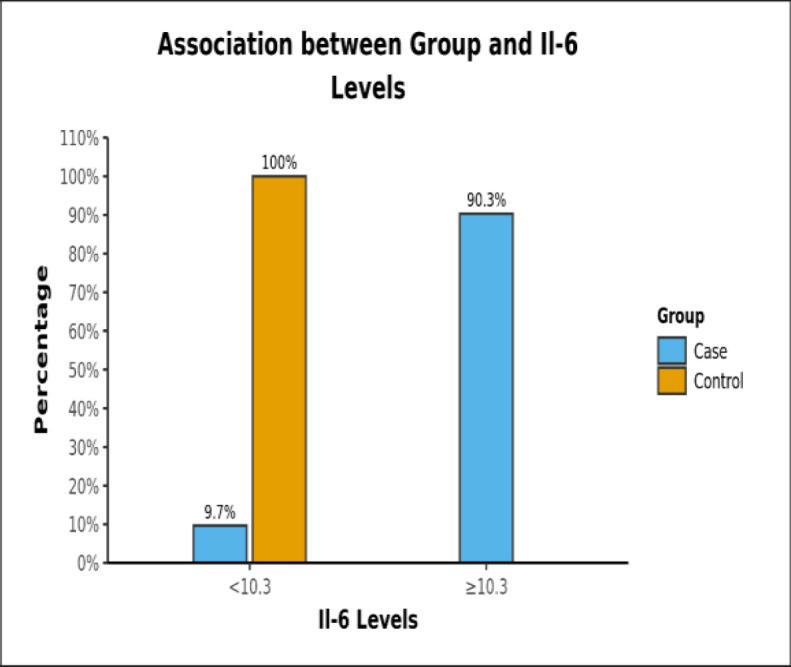
Percentage of two groups of IL-6 values in cases and controls

 The area under the ROC curve (AUROC) for Il-6 (<4.4) predicting Cases vs Controls was 0.941 (95% CI: 0.872 - 1), thus demonstrating excellent diagnostic performance. However, no significant association of IL-6 levels to the duration of disease or recurrence was found. There was a significant difference between the various groups in terms of distribution of TNF- bea levels (χ2 = 5.184, p = 0.039). Among Cases, 22.6% of the patients had TNF-beta <134.2 and 77.4% had TNF-beta ≥134.2. In the Control group 57.1% of the participants had TNF-beta <134.2 while 42.9% of the had TNF-beta levels ≥134.2 (Table 2).There was no significant association of TNF-β levels to the duration of disease or recurrence.

**Table 2:** Distribution of 2 groups of TNF-beta levels in cases and controls

**TNF-beta**	**Group**			**Fisher's Exact Test**	
	**Case**	**Control**	**Total**	**χ2**	**P Value**
<134.2	7 (22.6%)	8 (57.1%)	15 (33.3%)	5.184	0.039
≥134.2	24 (77.4%)	6 (42.9%)	30 (66.7%)		
Total	31 (100.0%)	14 (100.0%)	45 (100.0%)		

**Graph 2:**
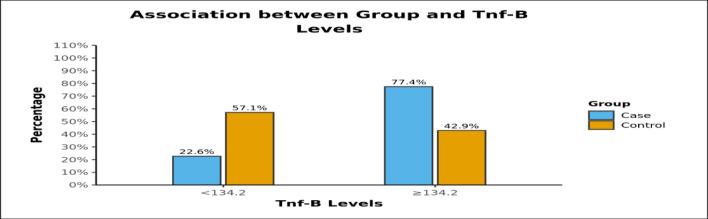
Distribution of 2 groups of TNF-beta levels in cases and controls

There was no significant association of TNF-β levels to the duration of disease or recurrence Distribution of 2 groups of TNF-beta levels in cases and controls. There was no significant difference between the various groups in terms of distribution of IFN-gamma levels (χ2 = 3.127, p = 0.156). 80.6% of the participants in case group had IFN-gamma levels: <16.32 and 19.4% of the had IFN-gamma levels: ≥16.32. None of the participants in the Control group had IFN-gamma levels: ≥16.32 (Table 3).

**Table 3:** Distribution of 2 groups of IFN-gamma in cases and controls

**IFN-gamma levels**	**Group**			**Fisher's Exact Test**	
	**Case**	**Control**	**Total**	**χ2**	**P Value**
<16.32	25 (80.6%)	14 (100.0%)	39 (86.7%)	3.127	0.156
≥16.32	6 (19.4%)	0 (0.0%)	6 (13.3%)		
Total	31 (100.0%)	14 (100.0%)	45 (100.0%)		

## Discussion

The endemicity of rhinosporidiosis may be due to geographical and climactic factors. It is well documented that contaminated water exposure is the major risk factor for the disease. The current study showed a universal pond bathing history in all patients but no significant association with disease duration and recurrence could be elicited. 

The period of latency for disease occurrence was also varied. A cessation of risk exposure didn’t negate disease recurrence attributing it more to the persisting residual microscopic remnants (even after extensive and meticulous excision with cauterization of the base) or inoculation of adjacent normal mucosa during surgery (6). 

**Fig 2:**
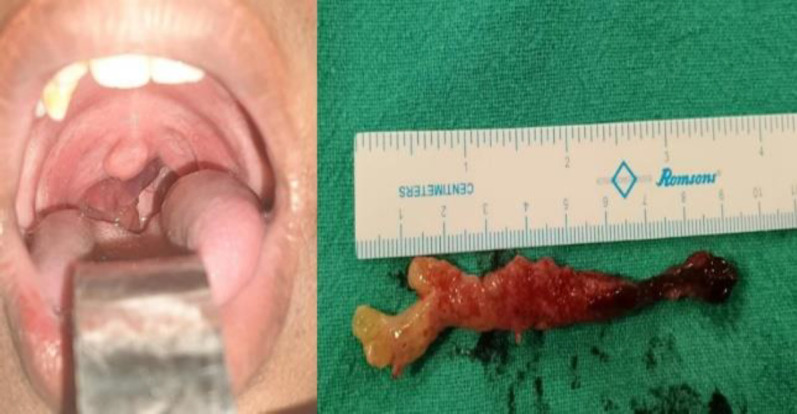
Rhinosporidial mass- oropharynx/ in toto excised naso-oropharyngeal mass

In our current study, 38.8% cases were recurrent disease who had already undergone surgery in the past. This is a considerably higher proportion compared to other studies like that of Sirshuk Dutta et al. (6) and Das et al. (7) where the recurrences were 13% and 8.33% respectively (Figure 2). 

The pattern of predominance of sites in our study was similar to other studies in literature. Saha J et al. 2011 (8) found that nasal presentation was the commonest and extra nasal manifestations were noted in fewer patients with more common involvement of oropharynx figure 1 than laryngopharynx with 1 patient presenting with lacrimal sac involvement. In the study by P. Karthikeyan et al. 2016, (9) the site of involvement was found to be nasal in majority (59.3%) cases, followed by nasopharyngeal involvement (21.8%) with only one patient having a disseminated disease.

In the present study, stalk attachment was in the nasal cavity in majority cases with common subsites being inferior turbinate and inferior meatus. This was of slight variation compared 

to study by P. Karthikeyan et al. 2016 (9) where the primary site of attachment was found to be inferior turbinate and septum and to the findings by Guru and Pradhan. 2014 (10) where the most common site of attachment was lateral wall of the nose, followed by septum and floor of nose. Endoscopic findings were found to be similar to that of CECT (Table 4).

**Table 4:** Comparison between Endoscopy- CECT- Intraoperative stalk attachments

**Stalk**	**Endoscopy**	**CECT**	**Intraoperative**
Nasal cavity	IT (77.4%)	IT (71.0%)	IT (87.1%)
Nasopharynx	IM (45.2%)	IM (38.7%)	IM (41.9%)
Oropharynx	Choana (9.7%) PPW (9.7%)	Choana (12.9%) PPW (22.6%)	Choana (16.1%) PPW (29.0%)
Oral cavity	Tongue (3.2%)	Tongue (3.2%)	Tongue (3.2%)

**Fig 3:**
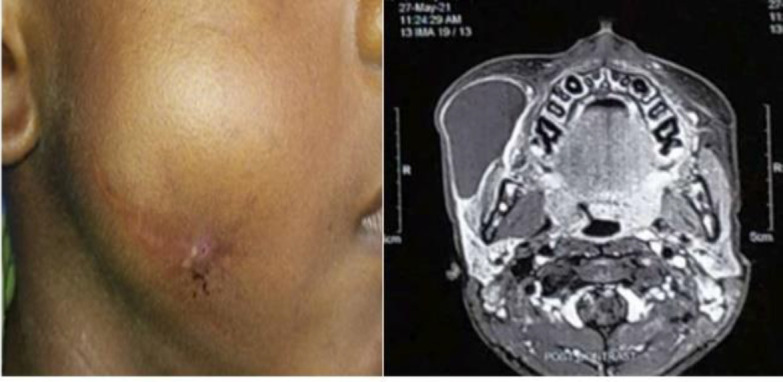
parotid ductocele- clinical/ radiology

Nasal cavity was in involved in majority cases with inferior turbinate and inferior meats being commonly involved sites. One patient had presented with a recurrent isolated parotid ductocele (Figure 2) which was soft, non-tender, unilocular, fluctuant. CT scan with dynamic contrast sialography revealed a cystic dilated part of stensen’s duct (left side) in the anterior part of the gland.Williams et al 2017 described a similar case of sialolith of parotid duct which on subsequent treatment and further histopathology of the ductocele revealed a causative factor for the obstruction of parotid duct to be rhinosporidiosis (11). Cutaneous presentation was present in 6.7% patients involving the forehead, upper arm, forearm, leg and ankle. Some presented as well-defined mass while others presented like bleeding, proliferative/ ulcerative lesions (Figure 3).

Changes in inflammatory markers and acute phase reactants- ESR, CRP, and Ferritin in rhinosporidiosis were evaluated in our study. Although there were marginal rises of inflammatory markers in patients, none were statistically significant. No significant association of their values to disease duration or recurrence was found which may be explained by the fact that these markers of inflammation are mostly associated with acute phase responses of inflammation and are most likely raised in the initial time period of the disease. Rhinosporidiosis is mostly a chronic infection with a lesser inflammatory response. So, it is less likely to have elevated inflammatory markers as seen in our study. 

There is no doubt that *Rhinosporidium seeberi* evokes immunological response in the host as suggested by the presence of anti-rhinosporidial antibodies. However, any protective role or association with disease characteristics are questionable. The various immunological aspects of the disease have been poorly explained in the literature as there are very few studies addressing them. Minimal information is available regarding any definite circulating antibodies, the extent of association of the common risk factor (contaminated water) to acquiring the infection and any inflammatory or immune response hosted by the body against the disease. In our study, a history of pond bathing habit was also present in other family members of the patient. Yet when it came to disease occurrence, only 12.9% patients gave a history of similar disease in the family. So, what is it that makes a patient susceptible to or protected from the disease is a question, the answer to which can be explained only through a thorough immunological assessment of the disease. This may have implications even in the treatment of the disease and explains the lack of definite medical therapy in rhinosporidiosis making surgery the gold standard treatment modality. R. A. Herr et al (14) in 1998 conducted a study on the immunolocalization of the antigens of the organism Rhinosporidium seeberi where they found antibodies that specifically recognized an internal electron-lucent layer situated immediately under the mature sporangial wall, absent in immature sporangia. This was the first report in which an antigenic material with a possible immunological role was detected and also explained why circulating antibodies were not detected in other studies which used endospores for antigen in their immunoassays. Arseculeratna et al. 2002 (5) studied the anti-rhinosporidial antibody levels in rhinosporidiosis patients and high-risk asymptomatic persons and found that the patients’ IgG titres were higher than Ig M titres (may be due to long duration of the disease). Their results indicated that an anti-rhinosporidial antibody response occurs in rhinosporidial patients and in asymptomatic persons exposed to the pathogen in the environment. These antibodies were not protective in rhinosporidiosis. de Silva et al.2001 **(**15) in their study indicated that cell-mediated immune responses develop in human rhinosporidiosis, along with some suppressed responses. All these studies recognized a definite immune response in rhinosporidiosis but there is no conclusive evidence in the literature regarding association with specific cytokines or immune markers. 

Since a cell-mediated immune response has been commonly implicated in rhinosporidiosis, in our study we have compared changes in cytokines of cell-mediated immunity- IL-6, IFN-γ, TNF-β- in patients with rhinosporidiosis and in controls without the disease. 

Our results showed a definite increase in IL-6 levels in patients compared to that of healthy controls, the result being statistically significant with a p-value <0.001. The area under the ROC curve demonstrated an excellent diagnostic performance. Also, there was a strong association of higher values of IL-6 with cases. However, on correlating the IL-6 levels with duration of disease and recurrence, no statistically significant results were obtained.

**Fig 4:**
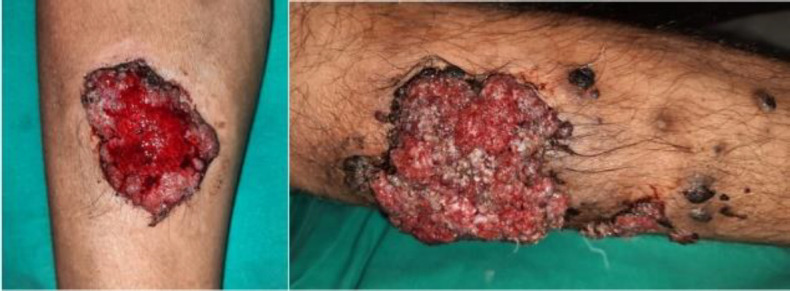
Cutaneous rhinosporidiosis- proliferative bleeding lesions

Similar findings were obtained for TNF-β levels which also showed a significant rise among cases. Even though the strength of association was only moderate as per the statistical evaluation, the comparison of TNF-β levels in cases and controls was found to be statistically significant (p-value= 0.039). There was no significant association of TNF-β levels to the duration of disease or recurrence. The results for IFN-γ levels, on the other hand, did not show a significant increase as in the case of IL-6 and TNF-β. No significant association was obtained between IFN-γ levels and duration or recurrence of the disease. The findings suggest that there is a role of cell-mediated immunity in rhinosporidiosis which needs to be explored further and cytokines like IL-6 and TNF-β may be part of the immune response (Figure 4). 

## Conclusion

Rhinosporidiosis is a disease of unresolved enigma in all aspects from taxonomy to treatment. Pond-bathing/ exposure to contaminated water is a major risk factor identified but its extent of risk and patient susceptibily is still not clearly defined. This requires an extensive immunological assessment of the disease which is limited in current literature. The decreased involvement of lymph nodes, notorious recurrence and chronicity despite presence of high antibody titre are all among the progressing list of questions surrounding the disease ^b^.

Being a chronic infection, rhinosporidiosis is not associated with significant increase in levels of inflammatory markers in the patients. The immune response elicited in the host is mostly cell-mediated. 

In our study we found a significantly increased levels of IL-6 and TNF-β (cytokines mostly associated with cell-mediated immunity) in patients with rhinosporidiosis but definite association with disease duration and recurrence was not elicited. The immunology of the disease seems to be the key in any new development in control and treatment aspects of the disease. This requires more studies and researches targeting the same. 
